# PGC-1α in osteoarthritic chondrocytes: From mechanism to target of action

**DOI:** 10.3389/fphar.2023.1169019

**Published:** 2023-04-06

**Authors:** Haochen Wang, Jianbang Su, Minghao Yu, Yang Xia, Yingliang Wei

**Affiliations:** ^1^ Department of Orthopedics, Shengjing Hospital of China Medical University, Shenyang, China; ^2^ Department of Clinical Epidemiology, Shengjing Hospital of China Medical University, Shenyang, China

**Keywords:** osteoarthritis, chondrocyte, PGC- 1α, metabolism, apoptosis, mitochondria

## Abstract

Osteoarthritis (OA) is one of the most common degenerative joint diseases, often involving the entire joint. The degeneration of articular cartilage is an important feature of OA, and there is growing evidence that the mitochondrial biogenesis master regulator peroxisome proliferator-activated receptor *γ* coactivator 1α (PGC-1α) exert a chondroprotective effect. PGC-1α delays the development and progression of OA by affecting mitochondrial biogenesis, oxidative stress, mitophagy and mitochondrial DNA (mtDNA) replication in chondrocytes. In addition, PGC-1α can regulate the metabolic abnormalities of OA chondrocytes and inhibit chondrocyte apoptosis. In this paper, we review the regulatory mechanisms of PGC-1α and its effects on OA chondrocytes, and introduce potential drugs and novel nanohybrid for the treatment of OA which act by affecting the activity of PGC-1α. This information will help to further elucidate the pathogenesis of OA and provide new ideas for the development of therapeutic strategies for OA.

## 1 Introduction

Osteoarthritis (OA) is the most common degenerative disease affecting the entire joint. The global age-standardized incidence rate (ASIR) of OA is increasing by 0.32% per year, an increase of about 9% in 28 years (Quicke et al., 2022), and its prevalence is estimated to double in the next 30 years. There are approximately 500 million people with OA worldwide, with an aging population and increasing obesity, more older adults will be disabled by OA (Hunter et al., 2020). OA is primarily characterized by pathological changes in articular cartilage, bone, synovium, ligaments, muscles and periarticular fat, resulting in joint dysfunction, pain, and functional limitations (Katz et al., 2021). Several drugs have shown therapeutic potential, but few have demonstrated the ability to arrest or slow the progression of OA (Abramoff and Caldera, 2020). Arthroplasty is an effective treatment for symptomatic end-stage OA, but suffers from a poor functional prognosis following functional surgery and a limited prosthetic life span (Glyn-Jones et al., 2015).

Healthy articular cartilage is a special type of hyaline cartilage, 2–4 mm thick, without blood or lymphatic vessels or nerves (Sophia Fox et al., 2009). Chondrocytes are the only cell type present in articular cartilage, infiltrating in the extracellular matrix (ECM) (Liu et al., 2018), which is rich in type II collagen and proteoglycans, and contributing to resisting compressive loads (Guilak et al., 2018). Loss of ECM and death of chondrocytes have been shown to be central features of articular cartilage degeneration (Pascarelli et al., 2015). Chondrocyte changes are an important feature in the pathogenesis of OA, but the mechanisms of cartilage destruction and loss of joint function in OA are not fully understood (Pascarelli et al., 2015).

Normal metabolism is closely related to chondrocyte physiology, in healthy joints, chondrocytes are in a state of physiological and metabolic homeostasis (Bai et al., 2019). Since articular cartilage is avascular, chondrocytes receive nutrients and oxygen primarily through low-rate diffusion through the ECM (Guilak et al., 2018; He et al., 2020). However, aerobic glycolysis is known to coexist with anaerobic glycolysis in normal chondrocytes (Hollander and Zeng, 2019). Chondrocytes take up glucose *via* specific glucose transporter proteins to maintain a stable energy metabolism. In addition, Cholesterol biosynthesis has been demonstrated in animal experiments to be elevated during normal growth plate cartilage formation in rats, and this effect has also been found in human cartilage (Aguilar et al., 2009; Bernstein et al., 2010; Yang et al., 2021a). Chondrocytes are also able to sense and transport lipoproteins to regulate lipid homeostasis in cartilage and maintain the ability of chondrocytes to perform their physiological functions (Villalvilla et al., 2013).

The mitochondrial biogenesis master regulator peroxisome proliferator-activated receptor *?* cofactor 1α (PGC-1α) is a 91 kDa transcription factor (Cheng et al., 2018) that is responsible for chondrocyte mitochondrial quality control (MQC), mtDNA expression, oxidative stress and metabolism, exerting a chondroprotective effect. Recent studies have shown that the expression and activity of PGC-1α are decreased in OA chondrocytes, which may be associated with degenerative changes in these cells (Wang et al., 2015).

This paper reviews the research on the effects of PGC-1α on OA chondrocytes, as well as the drugs and novel nanohybrid which may be useful for the treatment of OA by affecting PGC-1α activity, thus providing new ideas for further research into the pathogenesis, prevention and treatment of OA.

## 2 Chondrocyte cell death and OA

Cell death can occur in different ways and can be divided into programmed and non-programmed forms depending on the regulatory process involved (Yang et al., 2021b). Apoptosis is a highly-regulated, active process of programmed cell death and is involved in development. Apoptosis is an important process in the occurrence and development of OA, and the death of chondrocytes caused by apoptosis is positively correlated with the severity of OA (Komori, 2016). Recent studies have revealed several other types of cell death, including autophagy and ferroptosis (Sasaki et al., 2012). Ferroptosis, a newly discovered mode of programmed cell death caused by iron-dependent lipid peroxidation, has been shown to be involved in the pathogenic process of OA (Yao et al., 2021; Miao et al., 2022). In addition, most studies have proved that pyroptosis and necroptosis may be related to cartilage damage in OA (Riegger and Brenner, 2019; An et al., 2020), but the relationship between these special cell death modes and OA needs further research.

## 3 Regulatory pathways and molecules of PGC-1α in OA chondrocytes

### 3.1 AMPK/SIRT1 pathway

**FIGURE 1:**
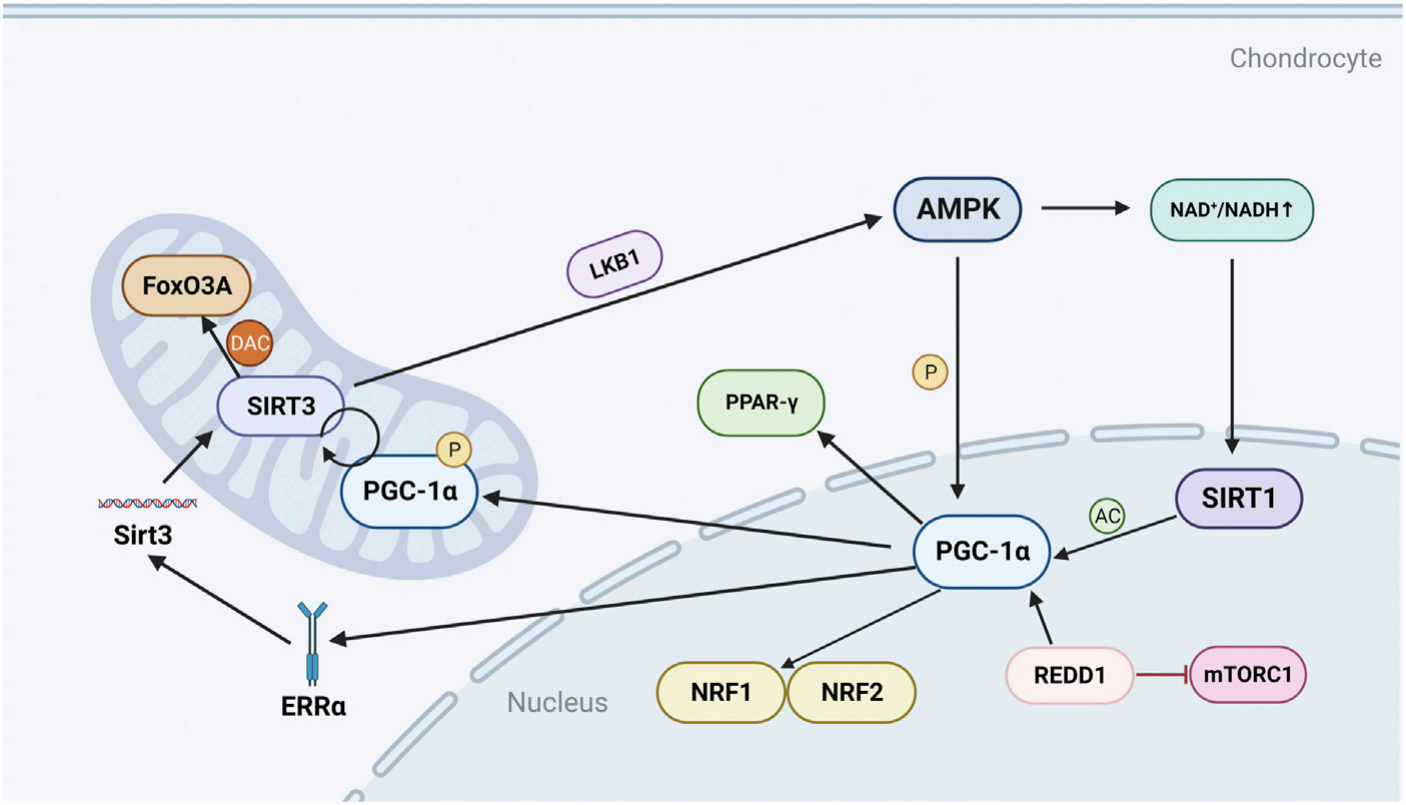
Regulatory mechanisms and molecules of PGC-1α in chondrocytes. AMPK can directly phosphorylate and activate PGC-1α, and can also activate SIRT1 by regulating NAD^+^/NADH, thereby acetylating and activating PGC-1α; PGC-1α can further promote the production of NRF1, NRF2 and TFAM; REDD1 is an endogenous inhibitor of mTOR and can regulate the transcriptional level of PGC-1α; PGC-1α can positively regulate SIRT3, and can also promote the expression of Sirt3 gene by mediating ERRα; SIRT3 can deacetylate FoxO3A in the mitochondrial matrix, and can also activate LKB1 to form a positive feedback loop to promote the expression of AMPK. P, phosphorylate; AC, acetylation; DAC, deacetylation.

AMP-activated protein kinase (AMPK) is a heterotrimeric complex comprising a catalytic subunit *a* and two regulatory subunits *ß* and *γ* (Yan et al., 2018). As an important regulator of energy homeostasis, AMPK responds to changes in the ratio of ATP to AMP by regulating metabolic enzymes to promote ATP production and inhibit ATP consumption (Herzig and Shaw, 2018). AMPK is a recognized upstream regulator of PGC-1α, which can directly affect the activity of PGC-1α through phosphorylation (Yao et al., 2023) (Figure 1). Silent information regulator 1 (SIRT1) is a histone deacetylase that maintains cartilage homeostasis by promoting chondrocyte proliferation, differentiation and survival, and upregulating genes important for cartilage function (Almeida and Porter, 2019). SIRT1 can affect the activity of PGC-1α through acetylation, and AMPK enhances SIRT1 activity by increasing cellular NAD^+^ levels, and further affects the activity of PGC-1α (Cantó and Auwerx, 2009; Zheng et al., 2020) (Figure 1). PGC-1α is a master regulator of mitochondrial biogenesis and function (Wang et al., 2015), and promotes mitochondrial transcription factor A (TFAM) expression by increasing downstream transcription of nuclear respiratory factor 1 (NRF1) and nuclear respiratory factor 2 (NRF2), thereby exerting the effects of TFAM in promoting mitochondrial biogenesis and mtDNA replication (Zhang et al., 2018a).

### 3.2 AMPK/SIRT3 pathway

Similar to SIRT1, silent information regulator 3 (SIRT3) is an NAD + -dependent protein deacetylase that is activated when translocated to mitochondria (Ansari et al., 2017). SIRT3 is an important regulator of chondrocyte energy metabolism, and SIRT3 enhances the antioxidant activity of superoxide dismutase (SOD2) to protect mitochondria from oxidative stress, and to repair mtDNA damage by deacetylating 8-oxoguanine DNA glycosylase-1 (OGG1) (Kincaid et al., 2013; Chen et al., 2021a).

In human knee chondrocyte experiments, it was found that AMPK can regulate the level and activity of SIRT3 (Chen et al., 2018). As mentioned above, AMPK can directly regulate the expression of PGC-1α, and PGC-1α can promote the expression of Sirt3 gene by mediating the binding of estrogen-related receptor-α (ERRα) to the Sirt3 promoter (Ansari et al., 2017) (Figure 1). PGC-1α can also promote SIRT3 expression directly or indirectly through the interaction of SIRT1 and NRF2 (Kong et al., 2010). In addition, SIRT3 also affects AMPK activity by promoting the expression of liver kinase B1 (LKB1) (Li et al., 2022). LKB1 is the main kinase that catalyzes the process of AMPK activation and energy production, and the activation of AMPK further promotes the expression of PGC-1α (Yao et al., 2023). Forkhead box class O 3 A (FoxO3A) is a transcription factor of the FOXO family, and like PGC-1α, limits cellular oxidative stress by upregulating antioxidant enzymes (Zhao et al., 2014; Almeida and Porter, 2019). SIRT3 deacetylates FoxO3A in the mitochondrial matrix and binds to mtDNA, promoting the upregulation of all mitochondria-encoded genes (Zhao et al., 2014). SIRT3 may also cooperate with FoxO3A to mediate the anti-oxidative stress effects of AMPK in chondrocytes.

In general, PGC-1α exerts its ability to resist oxidation and repair DNA damage through the AMPK/SIRT3 signaling pathway and its positive feedback mechanism (Chen et al., 2021a). SIRT3 may reverse mitochondrial dysfunction in OA through LKB1/AMPK signaling.

### 3.3 mTOR pathway

Mechanistic target of rapamycin (mTOR) is a serine-threonine protein kinase that forms two distinct complexes, mTOR complex 1 (mTORC1) and mTOR complex 2 (mTORC2). mTORC1 is highly sensitive to rapamycin and can be inhibited by tuberous sclerosis complex 1/2 (TSC1/2) (Yang et al., 2020). mTORC1 regulates the growth and proliferation of chondrocytes, osteoblasts and osteoclasts and is therefore critical for bone metabolism (Huang et al., 2015; Zhang et al., 2017a). Activation of mTORC1 can induce OA, whereas inhibition of mTORC1 by rapamycin to activate autophagy is protective in human chondrocytes (Zhang et al., 2017b). Furthermore, activation of mTORC1 was found to induce abnormal subchondral bone formation and promote OA in a mice model (Lin et al., 2019).

Mechanistically, the transcriptional level of PGC-1α is regulated by mTORC1. In skeletal muscle, mTORC1 regulates the oxidative capacity of skeletal muscle by changing the expression level of PGC-1α (Bentzinger et al., 2013). mTORC1 has also been shown to be involved in the decreased expression of PGC-1α due to Endoplasmic reticulum (ER) stress (Montori-Grau et al., 2022). In OA, development and DNA damage response-1 (REDD1), an endogenous inhibitor of mTOR, is reduced in articular cartilage (Figure 1). REDD1 controls mitochondrial biogenesis in chondrocytes by regulating the transcriptional level of PGC-1α (Alvarez-Garcia et al., 2017). Overall, although a direct link between PGC-1α and mTORC1 has been found in other tissues, the link in OA still needs to be further explored.

### 3.4 AMPK/PPAR-γ/PGC-1α pathway

Peroxisome proliferator-activated receptor-γ (PPAR-γ), a ligand-activated nuclear receptor, is an important target for the treatment of metabolic diseases (Wang et al., 2016). PPAR-γ-knockout mice with OA have decreased numbers of chondrocytes and increased expression of catabolic and inflammatory markers, so PPAR-γ can be used as a potential target for the treatment of OA (Vasheghani et al., 2015). PGC-1α is a transcriptional coactivator of PPAR-γ, and PPAR-γ stimulation promotes mitochondrial biogenesis by inducing PGC-1α (Zhang et al., 2021) (Figure 1). In an experiment on mice chondrocytes, it was found that the increased expression of PGC-1α and PPAR-γ could alleviate the increased expression of pro-inflammatory mediators and matrix metalloproteinases 13 (MMP-13) caused by high homocysteine (Ma et al., 2018a). The disordered expression of homocysteine may lead to mitochondrial dysfunction and oxidative stress in chondrocytes by inhibiting SIRT1 (Kalani et al., 2014). The experimental results confirmed that the disorder of homocysteine mitigated the activation of nuclear factor κB (NF-κB) pathway and reduced the expression of MMP-13, cyclooxygenase-2 (COX-2) and IL-8 in chondrocytes through AMPK/SIRT1/PGC-1α/PPAR-γ signal transduction (Ma et al., 2018a).

In addition, PPAR-γ also maintains the balance between catabolic and anabolic factors *in vitro* by regulating the mTOR/autophagy signaling pathway (Vasheghani et al., 2015). Given that OA is strongly associated with obesity and energy metabolism, the *in vivo* role of PPAR-γ in articular cartilage homeostasis requires further investigation.

## 4 PGC-1α reverses mitochondrial dysfunction in OA chondrocytes

### 4.1 PGC-1α promotes mitochondrial biogenesis in chondrocytes

**FIGURE 2:**
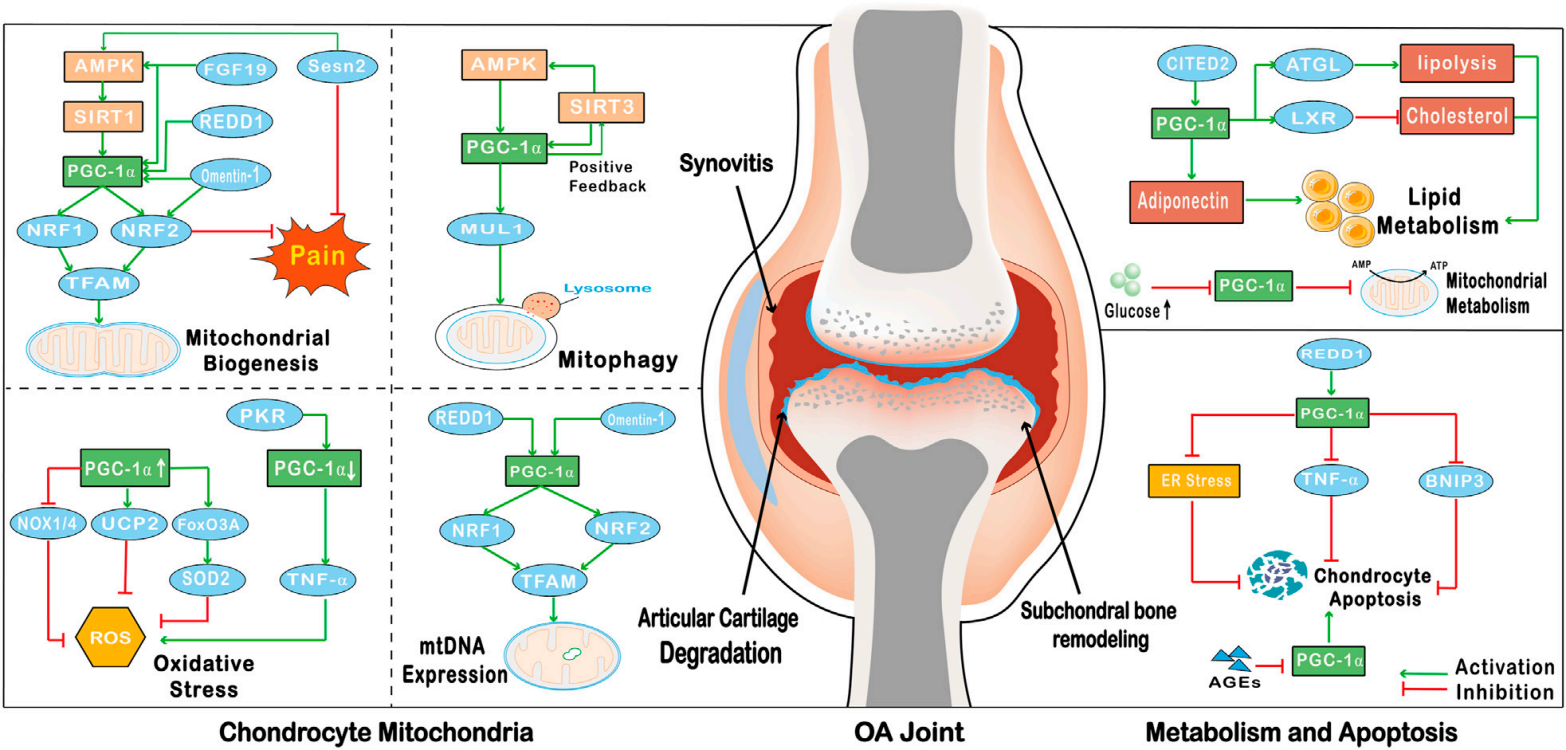
The effect of PGC-1α on OA chondrocytes. PGC-1α enhances the expression of NRF1, NRF2 and TFAM, and promotes the biogenesis of chondrocyte mitochondria; FGF19, REDD1 and Omentin-1 promote the biogenesis of chondrocyte mitochondria by promoting the expression of PGC-1α; Sesn2 reduces the pain of OA by promoting the expression of PGC-1α and NRF2; PGC-1α and SIRT3 form a positive feedback loop to promote the expression of autophagy factor MUL1 to promote mitophagy; the increase of PGC-1α expression can promote the expression of FoxO3A and UCP2 to inhibit the production of ROS; In addition, the increase of PGC-1α can also inhibit the expression of NOX1/4 to inhibit oxidative stress; On the contrary, PKR promotes the production of TNF-α by down-regulating the expression of PGC-1α to promote the production of ROS; REDD1 and Omentin-1 through affect the level of PGC-1α to promote the expression of mtDNA; PGC-1α promotes the catabolism of fatty acids and cholesterol by regulating the activity of ATGL and LXR, and CITED2 can also promote the expression of lipocalin by activating PGC-1α to regulate chondrocyte metabolism; High levels of glucose in OA chondrocytes can down-regulate the level of PGC-1α to inhibit mitochondrial metabolism; PGC-1α can be activated by REDD1 to down-regulate the level of inflammatory cytokine TNF-α to inhibit apoptosis. The effect of PGC-1α on inhibiting apoptosis can also be realized by inhibiting ER stress. In addition, the level of PGC-1α can be regulated by AGEs, and the decrease of PGC-1α level in chondrocytes can activate BNIP3 to promote apoptosis.

Mitochondrial biogenesis is a self-renewal process that continuously provides new mitochondria through growth and differentiation, and maintains mitochondrial homeostasis by clearing damaged mitochondria (Liu et al., 2022). In OA chondrocytes, the dysfunction of mitochondrial biogenesis is mainly manifested by decreased mtDNA content, mitochondrial mass, oxygen consumption, oxidative phosphorylation (OXPHOS), and intracellular ATP levels (Wang et al., 2021a). PGC-1α is activated through the AMPK/SIRT1 signaling pathway, and increased levels of PGC-1α can promote the expression of NRF1, NRF2 and TFAM, thereby promoting mitochondrial biogenesis (Zhang et al., 2018a) (Figure 2). Omentin-1, a newly discovered metabolically regulated adipokinine, promotes mitochondrial biogenesis in chondrocytes by enhancing the expression of PGC-1α, NRF1, and TFAM (Li et al., 2020). The cytokine fibroblast growth factors (FGFs) promote mitochondrial biogenesis in a similar mode to Omentin-1, and studies have shown that FGF19 enhances mitochondrial biogenesis and fusion through upregulation of AMPKα signaling (Kan et al., 2023). FGF19 increases the expression of p-AMPKα and PGC-1α, directly promoting mitochondrial biogenesis through the AMPK/SIRT1/PGC-1α axis (Herzig and Shaw, 2018) (Figure 2). Levels of PGC-1α were also associated with the molecule regulated in REDD1, which is a key factor in AMPK-induced transcriptional activation of PGC-1α in chondrocytes. REDD1 transcriptionally activates PGC-1α to promote mitochondrial biogenesis. Conversely, OA chondrocytes lacking REDD1 have reduced mitochondrial content, ATP levels, mitochondrial biogenesis, and expression levels of PGC-1α and TFAM (Sun et al., 2021a).

In addition, reduced mitochondrial biogenesis in chondrocytes may be associated with pain in OA. NRF2, produced by PGC-1α transcription, effectively relieves pain by regulating chondrocyte mitochondrial biogenesis (Sun et al., 2021a). Sestrins are a family of highly-conserved proteins induced by DNA damage and oxidative stress, and Sestrin2 (Sesn2) is a member of the Sestrin family. Overexpression of Sesn2 alleviates pain in monoiodoacetate-induced OA rats through AMPK/PGC-1α-mediated mitochondrial biogenesis (Sun et al., 2022) (Figure 2). Sesn2 also activates NRF2 with the help of PGC-1α expression and reduces ROS production, which may contribute to the relief of OA pain (Sun et al., 2022). Therefore, PGC-1α alleviates mitochondrial dysfunction in OA chondrocytes by promoting mitochondrial biogenesis, and can reduce the pain of OA.

### 4.2 PGC-1α promotes mitophagy in chondrocytes

Mitophagy is a specialized form of autophagy that functions to regulate the turnover of dysfunctional mitochondria and maintain mitochondrial homeostasis (Sun et al., 2021b). Additionally, mitophagy reduces the production of ROS and inhibits the activation of inflammatory factors in OA. The lack of mitophagy is an important factor in the development of OA disease, leading to chondrocyte death, imbalance of ECM homeostasis and cartilage degeneration (Hu et al., 2020).

In recent years, several studies have shown a link between PGC-1α and chondrocyte mitophagy. PGC-1α activates SIRT3 and regulates mitophagy through an AMPK/SIRT3 positive feedback loop (Chen et al., 2021a). In contrast, reduced expression levels of PGC-1α in OA chondrocytes activate parkin RBR E3 ubiquitin protein ligase (PRKN)-independent mitophagy *via* upregulation of Bcl-2/adenovirus E1B 19-kDa interacting protein (BNIP3), stimulating cartilage degradation and chondrocyte apoptosis (Kim et al., 2021). Furthermore, overexpression of PGC-1α suppresses FoxO3-mediated transcriptional activity, which further promotes the expression of various autophagy factors such as mitochondrial ubiquitin ligase 1 (MUL1) (Olmos et al., 2013) (Figure 2). One study has shown that dysregulation of mitophagy due to abnormalities in the AMPK/SIRT1/PGC-1α signaling pathway is a causative agent of sarcopenic obesity (Ryu et al., 2020), which may be associated with complications of OA, and therefore decreased levels of PGC-1α may induce and exacerbate OA by affecting mitophagy.

### 4.3 PGC-1α reduces oxidative stress in chondrocytes

Mitochondrial damage is associated with ROS production, which can be increased by a variety of factors including inflammatory cytokines, mechanical stress, and aging (Wang et al., 2021a). Increased ROS production and downregulation of SOD2 induce oxidative stress, which in turn leads to mitochondrial damage (Loeser et al., 2016). Loss of mitochondrial membrane potential in damaged mitochondria leads to decreased ATP production and increased mitochondrial membrane permeability, which is an important factor in chondrocyte senescence (Sun et al., 2021b). In addition, ROS induce oxidative stress and impair mitochondrial biogenesis, which has been shown to be associated with the development of chronic pain (Gao et al., 2022).

The activity of PGC-1α is inhibited in OA, leading to increased ROS production and oxidative stress. Double-stranded RNA-dependent protein kinase R (PKR) is an interferon-inducible kinase associated with cartilage degeneration that occurs in arthritic disease (Ma et al., 2018b). Increased PKR mediates activation of the inflammatory cytokine TNF-α by inhibiting the expression of PGC-1α, leading to increased oxidative stress and apoptosis in chondrocytes (Ma et al., 2018b). Activation of AMPK and SIRT1 modulates the activity of PGC-1α, thereby reducing oxidative stress and pro-metabolic responses in chondrocytes from OA patients (Wang et al., 2020), and relieving pain in patients with OA. PGC-1α and FoxO3A also limit cellular oxidative stress by upregulating antioxidant enzymes, including SOD2 and catalase (Zhao et al., 2014) (Figure 2). In addition, PGC-1α increases ATP production and reduces ROS production by altering the structure of the mitochondrial respiratory complex (Cunningham et al., 2007). PGC-1α also reverses the loss of chondrocyte phenotype by decreasing NADPH oxidase1/4 (NOX1/4) expression and increasing uncoupling protein 2 (UCP2) expression (Miao et al., 2017) (Figure 2). Consequently, PGC-1α has the ability to inhibit oxidative stress by reducing ROS production in chondrocytes, which is beneficial in delaying the further development of OA.

### 4.4 PGC-1α promotes the replication and gene expression of mtDNA in chondrocytes

Alterations in mitochondrial genetics are an important contributor to the development of OA. Increased ROS also leads to damage of mtDNA, causing a severe imbalance in redox and metabolic activity, which disrupts the homeostasis of articular cartilage (He et al., 2020). Reduced PGC-1α activity in OA is closely associated with decreased mitochondrial biogenesis and mtDNA content. PGC-1α activates NRF1 and NRF2 and promotes TFAM expression. Activation of PGC-1α, NRF1 and TFAM contributes to transcription and replication of mtDNA and the generation of new mitochondria (Kim et al., 2021). In addition, Omentin-1 promotes the expression of mtDNA, mRNA transcripts and mitochondrial proteins through the activation of PGC-1α (Li et al., 2020) (Figure 2). In conclusion, altered mitochondrial genetics is also a feature of OA, and like mitochondrial dysfunction, mtDNA damage in OA can be treated by affecting PGC-1α activity in chondrocytes.

## 5 Relationship between PGC-1α and abnormal metabolism of chondrocytes in OA

Abnormal chondrocyte metabolism is risk factor for OA and can have a direct systemic impact on the joints (Zheng et al., 2021). In the OA setting, the main components of the ECM, aggrecan and type II collagen, are reduced due to inhibition of chondrocyte synthetic activity (Wei et al., 2021). In OA cartilage, the rate of anaerobic glycolysis is increased and changes in key enzymes and glycolytic processes lead to the production and accumulation of excess lactic acid by chondrocytes, creating an acidic microenvironment (Maneiro et al., 2003). The acidic microenvironment has been shown to inhibit matrix synthesis in chondrocytes and potentially promote cartilage degeneration in OA (High et al., 2019). On the other hand, OA chondrocytes exhibit intracellular lipid deposition and the amount of lipid deposition is positively correlated with the severity of OA (Zheng et al., 2021).

Studies have shown PGC-1α regulates chondrocyte metabolism through the AMPK/SIRT1/PGC-1α signaling pathway, and has the ability to block chondrocyte pre-catabolic reactions (Zhao et al., 2014). PGC-1α is involved in the inhibition of advanced glycation end products (AGEs)-induced NF-κB activation and inflammatory cytokine-induced catabolic responses in chondrocytes (Li et al., 2021a; Yang et al., 2022). In contrast, decreased levels of PGC-1α in OA chondrocytes, possibly due to high levels of glucose promoting glycolysis and inhibiting oxidative phosphorylation, lead to impaired mitochondrial metabolism and trigger mitochondrial dysfunction (Minguzzi et al., 2018).

In addition to glucose metabolism, PGC-1α is also involved in the regulation of abnormal lipid metabolism in OA. PGC-1α is activated by SIRT1 in response to adipose triglyceride lipase (ATGL)-mediated increases in lipolysis (Khan et al., 2015). Activation of PGC-1α by PPAR-γ promotes Liver X receptors (LXR) expression, reduces cholesterol deposition on the joint surface, and maintains normal joint function and bone development (Ratneswaran et al., 2017). PGC-1α, which can be activated by Glu/Asp rich carboxy-terminal domain 2 (CITED2), plays a critical role in regulating load-induced adiponectin and inhibiting adiponectin expression in human infrapatellar fat pad-derived adipose stem cells/preadipocytes (Liu et al., 2019). In conclusion, PGC-1α is associated with obesity-induced OA and is involved in the regulation of abnormal glucose metabolism and lipid metabolism in OA chondrocytes (Ryu et al., 2020) (Figure 2).

## 6 Relationship between PGC-1α and chondrocyte apoptosis in OA

As mentioned above, increased ROS production and decreased SOD2 levels in OA chondrocytes lead to mitochondrial damage. Depolarization of mitochondria leads to the release of apoptotic factors such as cytochrome c (Cyt-c), apoptosis-inducing factor and capase-9 from the intermembrane space of mitochondria into the cytoplasm, resulting in apoptosis (Ansari et al., 2018). Numerous studies in recent years have revealed the relationship between autophagy, ER stress and chondrocyte apoptosis, indicating that autophagy may inhibit chondrocyte apoptosis by reducing ROS production through clearance of damaged mitochondria (Nugent et al., 2009). Increased ROS also induces ER stress, and sustained ER stress triggers the apoptotic pathway (Kim and Kim, 2018).

PGC-1α inhibits oxidative stress by reducing ROS production, blocking the activation of ER stress, and thus reducing chondrocyte apoptosis (Feng et al., 2019) (Figure 2). PGC-1α may also alleviate mitochondrial dysfunction in chondrocytes and reduce chondrocyte apoptosis by promoting mitochondrial biogenesis and accelerating the replication and expression of mtDNA. Conversely, decreased levels of PGC-1α may contribute to TNF-α induces chondrocyte apoptosis through the accumulation of oxidative stress *via* the PKR/p38 MAPK/p53/AKT/PGC-1α signaling pathway (Goldring, 2000). As an important mediator of OA, AGEs induce OA is by downregulating PGC-1α levels leading to increased oxidative stress, inflammation and apoptosis (Yu et al., 2022). Expression of REDD1 is regulated by PGC-1α, and Redd1^−/−^ mice have increased rates of apoptosis and increased indicators of cell death in knee chondrocytes (Alvarez-Garcia et al., 2017) (Figure 2). A study on homocysteine and OA showed that homocysteine dose-dependently inhibited the expression of AMPK/SIRT1/PGC-1α signaling in chondrocytes to promote chondrocyte apoptosis (Ma et al., 2018a). PGC-1α also has a role in promoting cartilage formation and differentiation, which may be achieved through interaction with the transcription factor sry-related high mobility group-box 9 (SOX9) (Kawakami et al., 2005). SOX9 is a key transcription factor in chondrocytes, co-expression of SOX9 and PGC-1α also induces the expression of other chondrogenic genes (Kawakami et al., 2005).

## 7 Potential drugs and strategies to treat OA by affecting the activity of PGC-1α

**FIGURE 3:**
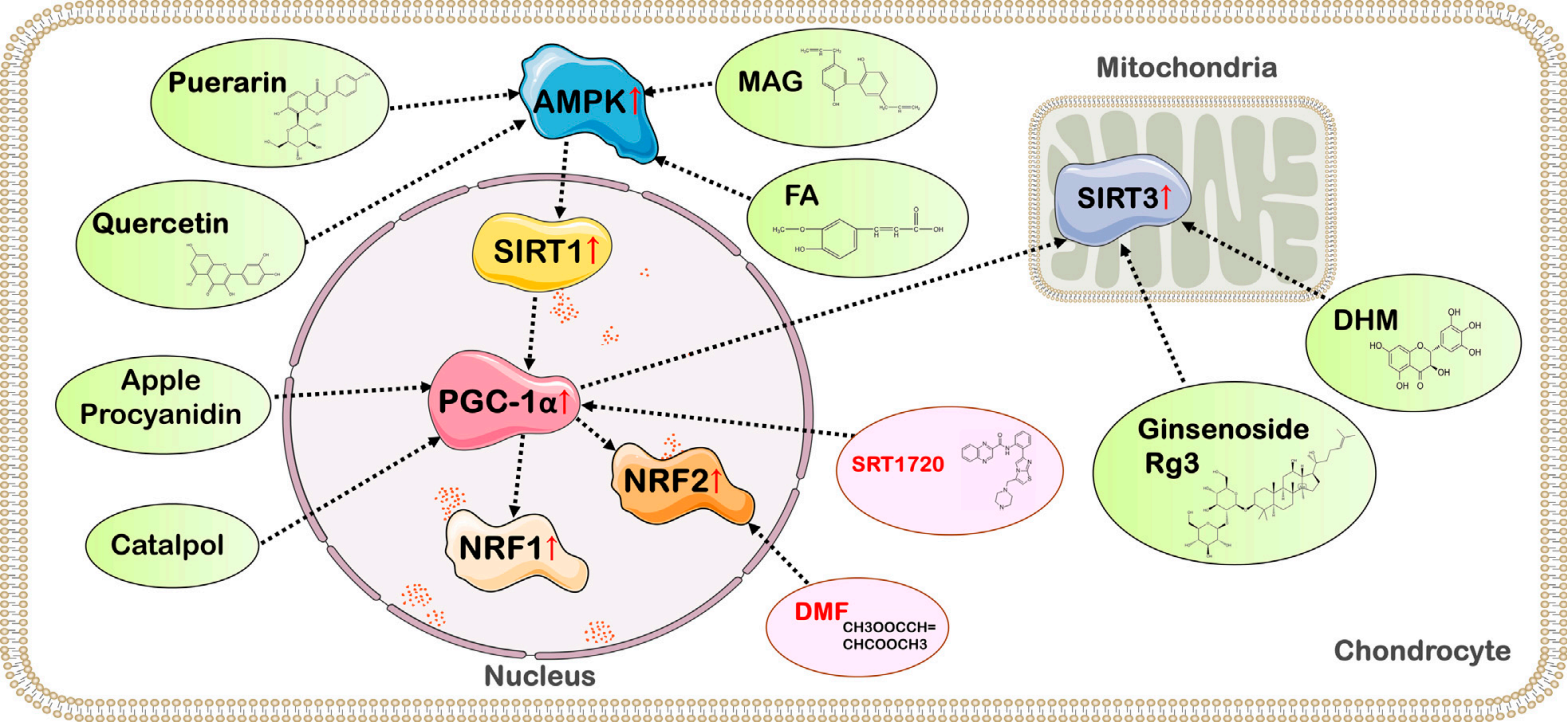
Targets of action of drugs with therapeutic effects on OA by affecting the activity of PGC-1α. Quercetin, FA, MAG, geraniin, and Br in RB@MPMW promote PGC-1α expression and delay the deterioration of OA by activating the AMPK/SIRT1/PGC-1α signaling pathway; Apple Procyanidins and catalpol can affect PGC-1α activity and ameliorate mitochondrial damage in OA chondrocytes; Ginsenoside Rg3 and DHM exert anti-inflammatory and chondrocyte homeostatic effects through the PGC-1α/SIRT3 pathway; SRT1720, an activator of SIRT1, exerts chondroprotective effects by activating SIRT1 and PGC-1α; DMF enhances NRF2 expression to promote mitochondrial biogenesis in chondrocytes.

PGC-1α is important to the mitochondrial function and energy metabolism of chondrocytes, and also delays the degeneration of articular cartilage by regulating the process of cell death. Improving the antioxidant capacity of chondrocytes and reversing mitochondrial dysfunction in OA chondrocytes by affecting PGC-1α levels may be a potential therapeutic strategy for OA. Therefore, it is necessary to develop new drugs and novel nanohybrid based on altering the activity of PGC-1α to improve the function of OA chondrocytes (Figure 3) (Table 1).

**TABLE 1:** Treatment of OA by affecting the activity of PGC-1α.

Potential drugs	Type	Mechanism	Effects	References
Quercetin	Natural Products	AMPK/SIRT1/PGC-1α	Mitochondrial biogenesis↑; Apoptosis↓	(Feng et al., 2019), (Qiu et al., 2018)
MAG	Natural Products	AMPK/SIRT1/PGC-1α; Inhibit IL-1β	Oxidative stress↓; Mitochondrial dysfunction↓	Liu et al. (2020)
FA	Natural Products	AMPK/SIRT1/PGC-1α; Inhibit IL-1β	ECM degradation↓; Oxidative stress↓	(Du et al., 2021), (Zhou et al., 2021)
Puerarin	Natural Products	AMPK/PGC-1α	Mitochondrial biogenesis↑	(Chen et al., 2021b), (Wang et al., 2018a), (Tak and Firestein, 2001)
Apple Procyanidin	Natural Products	Enhance PGC-1α; Inhibit TNF-α, MMP-13	Mitochondrial biogenesis↑; Integrity of mtDNA↑; SOD2↑	Masuda et al. (2018)
Ginsenoside Rg3	Natural Products	SIRT1/PGC1α/SIRT3; Inhibit TNF-α	ROS↓; Mitochondrial dysfunction↓	Ma et al. (2021)
Catalpol	Natural Products	Enhance phosphorylation of CREB	Mitochondrial biogenesis↑	Chen et al. (2022a)
DHM	Natural Products	AMPK/PGC-1α/SIRT3	Mitochondrial fusion↑; Mitophagy↑	(Wang et al., 2018b), (Mao et al., 2020)
DMF	Chemical Drugs	Enhance NRF2	IL-1β↓; Mitochondrial biogenesis↑, Type II collagen degradation ↓	(Gao et al., 2022), (Li et al., 2014)
SRT1720	Chemical Drugs	SIRT1/PGC-1α	LEF-1↓; Apoptosis↓; ECM expression↑	(Hu et al., 2021), (Liu et al., 2016)
RB@MPMW	Novel Nanohybrid	AMPK/SIRT1/PGC-1α	ROS↓; Apoptosis↓; Regulate energy metabolism	Xue et al. (2021)

### 7.1 Natural products

#### 7.1.1 Quercetin

Quercetin (3,3′,4′,5,7-pentahydroxyflavone) is a naturally occurring flavonoid found in many types of fruits and vegetables with anti-inflammatory, antioxidant, and anti-osteoporotic effects (Li et al., 2021b). In addition, quercetin alleviates cartilage degradation and thus OA by modulating chondrocyte autophagy (Lv et al., 2022), inflammation (Wang et al., 2021b), oxidative stress (Hu et al., 2019a), and apoptosis (Li et al., 2021b). Quercetin reduces the production of ROS and increases the expression levels of glutathione and glutathione peroxidase in OA rats. Quercetin also upregulates SOD and tissue inhibitor of metalloproteinase 1 while downregulating MMP-13 to attenuate oxidative stress and inhibit the degradation of cartilage extracellular matrix (Wei et al., 2019). Another animal study suggested that quercetin affects the characteristics and composition of the gut microbiota and metabolism of OA rats (Lan et al., 2021). Taken together, these data suggest that quercetin alleviates the deterioration of OA through multiple mechanisms.

In a rat model study of OA, researchers found that ROS production was reduced and mitochondrial biogenesis was improved after quercetin administration. In addition, the expression of p-AMPK, SIRT1, PGC-1α, NRF1, NRF2, and TFAM was also enhanced after quercetin application. Therefore, the antioxidative and mitochondrial dysfunction-reversing effects of quercetin may be achieved through the AMPK/SIRT1/PGC-1α signaling pathway (Qiu et al., 2018). Another animal study found that quercetin can also inhibit ER stress by activating the AMPK/SIRT1 signaling pathway, thereby inhibiting chondrocyte apoptosis, alleviating and eliminating articular cartilage degeneration, and thus treating OA (Feng et al., 2019). As mentioned above, quercetin may increase the expression of PGC-1α through the AMPK/SIRT1 signaling pathway, exerting the potential of treating OA in the mitochondrial pathway. However, most of the current studies on quercetin and OA use rat models, and further research is needed to explore its therapeutic potential and safety on human OA chondrocytes.

#### 7.1.2 Magnolol (MAG)

Magnolol (MAG) is extracted from a Chinese medicinal herb named Magnolia officinalis. A study on the link between MAG and OA showed that MAG exerted chondroprotective effects by inhibiting the production of inflammatory mediators as well as the degradation of OA chondrocyte proteoglycans and type II collagen (Hu et al., 2019b).

The effect of MAG on PGC-1α activity may be another mediator of its effects in OA. MAG increased PGC-1α expression in human chondrocytes in a dose-dependent manner and also alleviated IL-1β-induced mitochondrial dysfunction, oxidative stress and inflammation through the AMPK/SIRT1/PGC-1α signaling pathway and maintained the balance of ECM synthesis and catabolism in human chondrocytes (Liu et al., 2020). Therefore, MAG can be extracted and developed as a potential drug for OA.

#### 7.1.3 Ferulic acid (FA)

Ferulic acid (3-methoxy-4-hydroxycinnamic acid; FA), a phenolic substance widely found in plants (Klepacka and Łiu, 2006), is one of the most common natural products found in vegetable, and is an important active ingredient in many traditional Chinese medicines (Chaudhary et al., 2019). FA plays a role in the response to oxidative stress, inflammation, vascular endothelial damage, fibrosis, apoptosis and platelet aggregation, and is involved in the treatment of various diseases throughout the body (Chaudhary et al., 2019; Li et al., 2021c).

One of the mechanisms of FA treatment for OA is the inhibition of IL-1β-induced chondrocyte degeneration through the AMPK/SIRT1/PGC-1α signaling pathway. FA inhibits the production of IL-6, prostaglandin E_2_, nitrite, Collagen I, runt-related transcription factor 2, MMP-1, MMP-3 and MMP-13, suppresses oxidative stress, attenuates IL-1β-induced OA chondrocyte degeneration, and enhances expression of type II collagen and aggrecan through activation of PGC-1α (Du et al., 2021). Moreover, FA also prevents degradation of the ECM, inhibits the inflammatory response, and delays the onset and progression of OA (Zhou et al., 2021). The current study confirmed that FA, as a nutritional supplement for patients with OA, may have a potential therapeutic effect on OA by inhibiting inflammation (Du et al., 2021), but its clinical application value needs to be further explored.

#### 7.1.4 Puerarin

Puerarin (C_21_H_20_O_9_) is the main bioactive component isolated from the plant Pueraria montana var. Lobata (Zhou et al., 2014). Puerarin has antioxidant, anti-inflammatory, neuroprotective and anti-apoptotic activities, as well as lowering blood sugar and improving microcirculation. In OA, Puerarin has been shown to inhibit ECM degradation, relieve pain, and reduce cartilage destruction (Peng et al., 2019; Chen et al., 2021b; Li et al., 2021d). In addition, Puerarin was found to inhibit the production of the inflammatory factors IL-1β, IL-6 and TNF-α and increase type II collagen content in a rat OA model established by anterior cruciate ligament transection (Ma et al., 2020).

Although Puerarin has the ability to treat a variety of diseases, its molecular mechanisms and targets are not fully understood. There is evidence that the effect of Puerarin on OA rats may be achieved through the AMPK/PGC-1α pathway (Wang et al., 2018a). Experimental results in rat models have shown that puerarin can promote chondrocyte mitochondrial biogenesis through the AMPK/PGC-1α pathway, restore mitochondrial dysfunction in OA chondrocytes (Wang et al., 2018a), while reducing mechanical nociceptive hypersensitivity and cartilage damage in OA rats. In addition, in Puerarin-fed OA mice chondrocytes, the researchers found that Puerarin may inhibit the activation of NF-κB pathway and the degradation of ECM by regulating the level of NRF2 (Chen et al., 2021b). The activation of NF-κB pathway can enhance the inflammatory response of cells, leading to inflammatory injury and apoptosis (Tak and Firestein, 2001). Therefore, Puerarin is a dietary supplement with potential for the treatment of OA, but its role in the treatment of OA in humans needs further clinical verification.

#### 7.1.5 Apple procyanidin

Apple polyphenols (apple Procyanidin) are compounds of several polyphenols obtained from unripe apples and have been shown to have cardioprotective (Cicero et al., 2017), anti-inflammatory and anti-proliferative properties. An animal study showed that apple polyphenols reduced the severity of OA by inhibiting oxidative stress and the expression of TNF-α and MMP-13. Apple polyphenols also inhibited synovial inflammation in OA by enhancing cell proliferation and hyaluronic acid production (Kobayashi et al., 2022). Oral administration of apple polyphenols to mice was also shown to prevent articular cartilage degeneration caused by mitochondrial dysfunction. In addition, apple polyphenols were able to promote mitochondrial biogenesis and proteoglycan biosynthesis in chondrocytes and enhance aggrecan upregulation in primary chondrocytes, which may be achieved by affecting PGC-1α activity (Masuda et al., 2018). Mechanistically, apple polyphenols enhance mitochondrial dehydrogenase activity and mitochondrial DNA copy number, and promote PGC-1α expression to promote mitochondrial biogenesis. Apple polyphenols also improve mitochondrial depolarization impaired by SOD2 loss by affecting PGC-1α activity (Masuda et al., 2018). These results demonstrate that apple polyphenols may be food components with effects on maintaining joint cartilage health.

#### 7.1.6 Ginsenoside Rg3

Ginsenoside Rg3 is a steroidal saponin isolated from ginseng and is the main active component of ginseng (Liu et al., 2021). Recent studies have shown that Rg3 has various biological activities such as anti-inflammatory and anti-cancer (Xia et al., 2022). In the experimental model of human OA aging chondrocytes, Ginsenoside Rg3 has the effect of anti-aging and protecting cartilage (So et al., 2013). A new study suggests that activation of the SIRT1/PGC-1α/SIRT3 pathway by Rg3 inhibits TNF-α-induced cyclophilin D acetylation, reduces mitochondrial dysfunction and ROS accumulation, thereby ameliorating TNF-α-induced apoptosis (Ma et al., 2021). Additionally, Rg3 suppresses TNF-α-stimulated p38 MAPK phosphorylation and NF-κB activation through SIRT1/PGC-1α/SIRT3 signaling, and inhibits TNF-α-induced increases in production of IL-8 and MMP-9 (Ma et al., 2021). The SIRT1/PGC-1α/SIRT3 pathway may be the main mechanism *via* which Rg3 acts to inhibit expression of the inflammatory cytokine TGF-α, so Ginsenoside Rg3 has the potential to alleviate the inflammatory response and cartilage degeneration in OA patients.

#### 7.1.7 Catalpol

Catalpol, an active ingredient from the traditional Chinese herbal medicine Di-Huang (Rehmannia glutinosa Libosch or Chinese foxglove), has potential antioxidant and hypoglycemic effects (Bhattamisra et al., 2021). Previous studies have shown that in chondrocytes, catalpol can attenuate IL-1β-induced inflammatory response and apoptosis in rat chondrocytes by inhibiting the NF-κB pathway (Zeng et al., 2019). In addition, catalpol was also found to enhance mitochondrial biogenesis in human chondrocytes through a dose-dependent increase in phosphorylation of cAMP/response element-binding protein (CREB) to promote the expression of PGC-1α, NRF1 and TFAM (Chen et al., 2022a). In addition, catalpol improves mitochondrial ATP production, Cyt-c oxidase activity, and respiratory rate. Although the current study shows that catalpol has chondroprotective effect, further detailed mechanism is needed before it can be used for the treatment and prevention of OA.

#### 7.1.8 Dihydromyricetin (DHM)

DHM is a flavonoid with modulatory metabolic, anti-inflammatory, antioxidant, antitumor, pyroptosis-reducing and cardioprotective effects (Zhang et al., 2018b; Cheng et al., 2020; Sun et al., 2020). DHM increases SIRT3 and PGC-1α levels in a dose-dependent manner through the AMPK/SIRT3/PGC-1α signaling pathway, improves antioxidant capacity and mitochondrial fusion in chondrocytes, increases the levels of aggrecan and type II collagen, maintains chondrocyte homeostasis and prevents chondrocyte degeneration (Wang et al., 2018b). The level of PGC-1α is positively correlated with the activity of SIRT3, and DHM also activates SIRT3 by increasing the level of PGC-1α to regulate the mitochondrial dynamics and mitophagy in chondrocytes (Mao et al., 2020), providing a new idea for the treatment of OA.

### 7.2 Chemical drugs

#### 7.2.1 Dimethyl fumarate (DMF)

Dimethyl fumarate (DMF) is a fumarate ester with cytoprotective, anti-inflammatory and antioxidant properties, induces protein succinylation, which leads to inactivation of cysteine-rich proteins (Saidu et al., 2019). As mentioned above, PGC-1α can activate NRF2 to regulate mitochondrial biogenesis, and DMF mainly plays a role by regulating the level of NRF2. Experimental animal studies have shown that DMF improves renal injury and cognitive deficits by activating NRF2 to prevent ferroptosis (Yang et al., 2021c; Yan et al., 2021). For OA, DMF inhibits IL-1β expression by activating NRF2, thereby attenuating destabilization of the medial meniscus-induced OA in mice (Chen et al., 2022b). Furthermore, DMF induced mitochondrial biogenesis and attenuated pain-related behaviors in a rat model of OA by activating NRF2 (Gao et al., 2022). In human chondrocytes, experiments have shown that DMF can inhibit OA-induced degradation of type II collagen, suggesting that DMF treatment may be a potential chondroprotective strategy (Li et al., 2014).

#### 7.2.2 SRT1720

SRT1720 is a synthetic compound that activates SIRT1((Minor et al., 2011; Svensson et al., 2015)) and has been implicated in the regulation of neurogenesis (Iwata et al., 2020) and angiogenesis (Dadwal et al., 2021) through activation of SIRT1. It has also been shown that SRT1720 can reduce pain due to bone cancer (Li et al., 2019) and intervertebral disc degeneration (Zhang et al., 2020) by activating SIRT1. In OA, intraperitoneal injection of SRT1720 has been shown to slow the progression of experimental OA in mice (Nishida et al., 2018). SRT1720 also exerts a chondroprotective effect by increasing the level of SIRT1 to regulate the expression of lymphoid enhancer-binding factor 1 (LEF-1) and related inflammatory factors in OA (135). In addition, an animal study on rabbit chondrocytes suggested that SRT1720 inhibited OA cell apoptosis by activating SIRT1 and PGC-1α, thereby protecting chondrocytes and promoting the expression of cartilage matrix (Liu et al., 2016). In conclusion, the mechanism of action of SRT1720 is not fully understood so far, but its chondroprotective effect provides a new strategy for the treatment of OA.

### 7.3 Novel nanohybrid

#### 7.3.1 Cartilage-targeting peptide-modified dual-drug delivery nanoplatform with NIR laser (RB@MPMW)

Cartilage-targeted drug delivery is an effective strategy for the treatment of OA, and the design of novel drug delivery systems has been an important direction of OA treatment research in recent years (Xiong et al., 2021). As mentioned above, PGC-1α has a role in regulating mitochondrial function and chondrocyte energy metabolism. Cartilage-targeted peptide-modified near-infrared laser dual drug delivery nanoplatform is a recently reported novel material for the treatment of OA that regulates chondrocyte energy metabolism through sustained phosphorylation of AMPK by bilirubin (Br) (Idelman et al., 2015; Vítek, 2020) and thus promotes activation of PGC-1α. The platform loads rapamycin in mesopores, Br on the metal organic framework shell, and type II collagen-targeting peptide bound to the above nanocarrier surface. Near-infrared laser stimulation releases both rapamycin and Br drugs continuously. Br acts as an activator of AMPK and activates PGC-1α by activating the AMPK/SIRT1/PGC-1α signaling pathway, exerting the ability of PGC-1α to scavenge ROS and inhibit apoptosis (Xue et al., 2021). Activation of PGC-1α also inhibits the action of the inflammatory cytokine IL-β and regulates the energy metabolism of chondrocytes (Xue et al., 2021), thus preventing cartilage degeneration *in vivo*.

## 8 Discussion

Results of recent studies suggest that PGC-1α, as a master regulator of mitochondrial biogenesis and metabolism, may be a candidate target for the treatment of OA. PGC-1α is activated by multiple pathways and signaling molecules in chondrocytes to regulate mitochondrial biogenesis, mitophagy, and mtDNA replication and expression. In addition, PGC-1α can inhibit oxidative stress and chondrocyte apoptosis to maintain cartilage homeostasis. PGC-1α is also involved in the regulation of abnormal energy metabolism and lipid metabolism in OA chondrocytes, and inhibits the catabolic reactions of chondrocytes. The chondroprotective effect of PGC-1α has also been demonstrated in drug experiments, and multiple effectors have shown beneficial effects in experimental models of OA by activating PGC-1α in chondrocytes. However, the value of target-activating PGC-1α effectors and novel nanohybrids in clinical applications needs further investigation. The role of PGC-1α on other metabolic pathways of chondrocytes and other pathological changes of OA is still unclear. Therefore, in the future experimental research and clinical treatment of OA, PGC-1α and its related pathways and regulatory molecules deserve special attention.

## Glossary

PGC-1α: peroxisome proliferator-activated receptor *γ* cofactor 1α
mtDNA: mitochondrial DNA
OA: osteoarthritis
ASIR: age-standardized incidence rate
ECM: extracellular matrix
MQC: mitochondrial quality control
AMPK: AMP-activated protein kinase
SIRT1: silent information regulator 1
TFAM: mitochondrial transcription factor A
NRF1: nuclear respiratory factor 1
NRF2: nuclear respiratory factor 2
SIRT3: silent information regulator 3
SOD2: superoxide dismutases 2
OGG1: 8-oxoguanine DNA glycosylase-1
ERRα: estrogen-related receptor-α
LKB1: liver kinase B1
FoxO3A: forkhead box class O 3A
mTOR: mechanistic target of rapamycin
mTORC1: mTOR complex 1
mTORC2: mTOR complex 2
TSC1/2: tuberous sclerosis complex 1/2
ER: endoplasmic reticulum
REDD1: regulated in development and DNA damage response 1
PPAR-γ: peroxisome proliferator-activated receptor-γ
MMP-13: matrix metalloproteinase-13
NF-κB: nuclear factor κB
COX-2: cyclooxygenase-2
OXPHOS: oxidative phosphorylation
FGFs: fibroblast growth factors
Sesn2: Sestrin2
PRKN: parkin RBR E3 ubiquitin protein ligase
BNIP3: Bcl-2/adenovirus E1B 19 kDa interacting protein 3
MUL1: mitochondrial ubiquitin ligase 1
PKR: double-stranded RNA-dependent protein kinase R
NOX1/4: NADPH oxidase1/4
UCP2: uncoupling protein 2
AGEs: advanced glycation end products
ATGL: adipose triglyceride lipase
LXR: liver X receptor
CITED2: CBP/p300 interacting transactivator with Glu/Asp rich carboxy-terminal domain 2
Cyt-c: cytochrome c
SOX9: sry-related high mobility group-box 9
MAG: magnolol
FA: ferulic acid
CREB: cAMP/responsive element-binding protein
DHM: dihydromyricetin
DMF: dimethyl fumarate
LEF-1: lymphoid enhancer-binding factor 1
RB@MPMW: cartilage-targeting peptide-modified dual-drug delivery nanoplatform with near-infrared laser
Br: bilirubin
